# Toward an operative diagnosis of fussy/picky eating: a latent profile approach in a population-based cohort

**DOI:** 10.1186/1479-5868-11-14

**Published:** 2014-02-10

**Authors:** Anne Tharner, Pauline W Jansen, Jessica C Kiefte-de Jong, Henriette A Moll, Jan van der Ende, Vincent WV Jaddoe, Albert Hofman, Henning Tiemeier, Oscar H Franco

**Affiliations:** 1 Department of Epidemiology, Erasmus University Medical Center, Rotterdam, The Netherlands; 2 Department of Child and Adolescent Psychiatry/Psychology, Erasmus University Medical Center, Rotterdam, The Netherlands; 3 Department of Pediatrics, Erasmus University Medical Center, Rotterdam, The Netherlands; 4 Generation R Study Group, Erasmus University Medical Center, Rotterdam, The Netherlands

**Keywords:** Fussy eating, Children, Latent profile analysis, Population-based cohort study, Dietary intake, BMI, Child and family characteristics

## Abstract

**Background:**

Definitions and assessment methods of fussy/picky eating are heterogeneous and remain unclear.

We aimed to identify an eating behavior profile reflecting fussy/picky eating in children and to describe characteristics of fussy eaters.

**Methods:**

Eating behavior was assessed with the Child Eating Behavior Questionnaire (CEBQ) in 4914 4-year olds in a population-based birth cohort study. Latent Profile Analysis (LPA) was used to identify eating behavior profiles based on CEBQ subscales.

**Results and discussion:**

We found a “*fussy*” eating behavior profile (5.6% of children) characterized by high food fussiness, slowness in eating, and satiety responsiveness in combination with low enjoyment of food and food responsiveness. *Fussy* eaters were more often from families with low household income than non-fussy eaters (42% vs. 31.8% respectively; *Χ*^*2*^(1) = 9.97, *p* < .01). When they were 14 months old, *fussy* eaters had a lower intake of vegetables (*t* [3008] = 2.42, *p* < .05) and fish (*t* [169.77] = 2.40, *p* < .05) but higher intake of savory snacks (*t* [153.69] = -2.03, *p* < .05) and sweets (*t* [3008] = -2.30, *p* < .05) compared to non-fussy eaters. Also, *fussy* eaters were more likely to be underweight at 4 years of age (19.3%) than non-fussy eaters (12.3%; *Χ*^*2*^(1) = 7.71, *p* < .01).

**Conclusions:**

A distinct *fussy* eating behavior profile was identified by LPA, which was related to family and child characteristics, food intake, and BMI. This behavior profile might be used in future research and the development of interventions.

## Background

Fussy or picky eating is an increasing concern in pediatric care because it is related to a higher risk of underweight, low vegetable consumption, not meeting age-specific dietary recommendations, eating disorders and mother-child conflicts [1-6]. However, comparability of previous research on fussy/picky eating has been limited by the use of inconsistent definitions and assessment methods [6].

Definitions of fussy/picky eating usually include rejection of specific familiar foods and new foods (food neophobia), but also extend to inadequate amounts of food consumed, or rejecting certain food textures [6]. One of the methods to assess fussy/picky eating applied by previous studies is to ask mothers whether or not their child is a fussy or picky eater, for example by indicating on a single 5-point scale whether the child is ‘never’ to ‘always’ a picky eater [7,8] or similarly, whether their child is a very picky eater, a somewhat picky eater or not a picky eater [3]. Another method classifies children as “picky eaters” if mothers indicated that they always or often displayed difficult eating behavior as assessed by three items (e.g. “Refuses to eat.”) [9,10].

In addition, several questionnaires are available that include scales about problematic eating, fussy/picky eating and/or food neophobia: The *Child Feeding Questionnaire*[11] assesses pickiness on a continuous scale score which has been used to specify a group of “picky eaters” based on a median split cut-off [2,12]. The *Children’s Eating Behavior Inventory*[13] assesses problematic eating behavior on a continuous scale, with higher scores reflecting more problematic eating behavior. The *Child Eating Behavior Questionnaire* (CEBQ) [14] consists of four scales assessing “food approach” behaviors (i.e. emotional overeating, food responsiveness, enjoyment of food, and desire to drink) and four scales measuring “food avoidance” behaviors (i.e. emotional undereating, satiety responsiveness, slowness in eating and food fussiness). Previous studies using the CEBQ have mainly assessed fussy/picky eating with a continuous score on the “food fussiness” subscale consisting of 6 items (e.g. “Refuses to eat new food at first”) [1,15]. Finally, some studies have combined items from different questionnaires to assess picky eating on a continuous scale and defined a “picky eater” group based on the specific behaviors being sometimes or always present [16,17].

Although many methods have been used, it remains unclear how to define fussy/picky eating. The large heterogeneity of assessment methods and definitions used in previous studies indicates that fussy/picky eating is a complex phenomenon and that there is a need for a more applicable measure [6]. Studies reporting that food fussiness/pickiness is related to a variety of other problematic eating behavior [14,17,18] also suggest that fussy/picky eating might be best captured in a behavioral pattern that takes into account several eating behaviors in addition to the unwillingness to eat certain types of food. In this study we therefore aimed to identify a behavioral profile that reflects fussy/picky eating in children using the different eating behavior styles assessed with the CEBQ [14] in a large birth cohort in the Netherlands, and to describe the characteristics of the identified profiles. The CEBQ is one of the most comprehensive existing questionnaires to assess children’s eating behavior. It covers a wide range of behaviors that have been described previously in relation to fussy eaters, such as refusal to eat new foods, but also addresses more general problematic eating behavior (food avoidance) as well as the opposite, i.e. food approaching behaviors. We hypothesized that in order to better reflect the complexity of fussy/picky eating behavior, a profile would comprise a combination of low scores on the food approach scales of the CEBQ and high scores on the food avoidance scales. In addition to the specific eating behavioral characteristics, we hypothesized that fussy/picky eating behavioral profile would relate to more problematic parental feeding behavior and altered infant food intake. Differences between fussy and non-fussy eaters in several child and family characteristics were also explored.

## Methods

### Subjects

This study was embedded within the Generation R Study, a population-based cohort from fetal life onwards [19,20]. All pregnant women living in Rotterdam, the Netherlands, with an expected delivery date between April 2002 and January 2006 were invited to participate (participation rate: 61%). The study was conducted in accordance with the guideline proposed in the World Medical Association Declaration of Helsinki and has been approved by the Medical Ethics Committee of the Erasmus Medical Center, Rotterdam. More detailed information about the study design can be found elsewhere [19]. Written informed consent was obtained from all adult participants. Full consent for the preschool phase of the Generation R Study was obtained from parents of 7295 children. For 2315 children with postnatal consent for participation, the CEBQ was missing due to non-response. Additionally, we excluded 66 children due to partial missing information on the CEBQ. This resulted in a sample of 4914 children who were included in at least one analysis (67% of all children with full postnatal consent). Dietary data at 14 months were available for 3010 (61%) of these children.

### Measures

#### ***Children’s eating behavior***

Eating behavior was assessed when the child was four years old by a Dutch version of the CEBQ [1,14]. The CEBQ consists of 35 items scored on a 5-point Likert scale from 1 ‘never’ to 5 ‘always’. Items are assigned to eight subscales, i.e. Emotional Overeating (EOE), Food Responsiveness (FR), Enjoyment of Food (EF), Desire to Drink (DD), Emotional Undereating (EUE), Satiety Responsiveness (SR), Food Fussiness (FF) and Slowness in Eating (SE). Examples of items are “My child loves food” (EF), “Even if my child is full up, s/he finds room to eat his/her favorite food” (FR), “My child refuses to eat new food at first” (FF) and “Eats slowly”(SE). Subscales represent two dimension, i.e. “food approach” (EOE, EF, FR, DD) and “food avoidance” (EUE, SR, FF, SE). All items are listed in Additional file 1: Table S1. In accordance with earlier studies [1], scale scores were corrected for the number of endorsed items ((raw score/number of endorsed items)* maximum number of items), with a maximum of 25% missings allowed. The continuous CEBQ scale scores were expressed as z-scores to facilitate effect size comparison between scales. Higher scores on each subscale indicate the respective behavior is more evident. The CEBQ has good psychometric properties, such as good internal consistency, concurrent validity with actual eating behavior, test-retest reliability, and stability over time [10,14,21].

To confirm the good psychometric properties of the CEBQ in our large, population-based sample, we conducted exploratory and confirmatory factor analyses (EFAs and CFAs) in Mplus [22,23]. Geomin rotation was used in the EFA. To increase power but keep the introduced uncertainty limited, we allowed for a maximum of 2 missing items of the 35 CEBQ items. Five items had to be reverse coded (item 14, 21, 23, 24, and 25) to construct ascending subscale scores. Exploratory factor analysis of the 35 CEBQ items (*N* = 4914) identified eight factors with an Eigenvalue of 1 or higher, explaining a total of 68% of the variance (Additional file 1: Table S1). This 8-factor solution was supported by examination of the scree-plot. The findings almost completely matched the expected structure of the subscales as defined by Wardle and colleagues [14]. Model fit indices indicated a good fit of this solution (comparative fit index CFI 0.968; Tucker-Lewis index TLI 0.944). An additional CFA confirmed the general factor structure although fit indices were somewhat lower (CFI 0.897, TLI 0.884). As expected, subscales representing food avoidance (SR, FF, SE) correlated positively with one another, but negatively with subscales representing food approach (FR, EF) (Additional file 2: Table S2). FR and EF correlated positively. By contrast, emotional undereating and overeating were positively correlated. No clear correlational pattern emerged for desire to drink, which was positively correlated with EOE and FR but not correlated with any other scale. Internal consistency of the original subscales was good with Cronbach’s α ≥ .74 (EOE Cronbach’s α = 0.85; FR Cronbach’s α = 0.84; EF Cronbach’s α = 0.89; DD Cronbach’s α = 0.88; EUE Cronbach’s α = 0.78; SR Cronbach’s α = 0.74; SE Cronbach’s α = 0.74; FF Cronbach’s α = 0.89).

#### ***Child characteristics***

Information about child gender and birth weight were obtained from midwife and hospital registries. Ethnicity of the child was based on country of birth of both parents, and categorized into Western and Non-Western. If ethnicity of both parents did not correspond, the child was assigned the ethnicity of the mother [24]. Daycare attendance was assessed by questionnaire when the child was three years old. Trained staff of the municipal Child Health Centers obtained children’s growth characteristics as part of a routine health care program in the Netherlands. Visits take place regularly during the first years of the child’s life. The current study uses data from the visit scheduled around the fourth birthday. Weight was measured by a mechanical personal scale (SECA^®^) while children were wearing underwear only. Height was measured bare-footed in standing position by a Harpenden stadiometer (Holtain Limited^®^). Body Mass Index (BMI) was calculated as weight/height^2^ (kg/m^2^). BMI is expressed in age- and sex-specific standard deviation scores, calculated using the Dutch reference curves [25] in the Growth Analyser program [26]. International age- and sex-specific cut-offs were used to classify children into four different weight groups: underweight [27], normal weight, overweight and obese [28].

#### ***Family characteristics***

Parental BMI was calculated as weight/height^2^ (kg/m^2^). Ethnicity, marital status (single or not single), educational level, family income, smoking habits during pregnancy and history of eating disorders were assessed by postal questionnaire. Maternal educational level was coded as high (some college or university education) or not high. For income, we used 2200€ a month as a cut-off to indicate below modal household income. Maternal smoking during pregnancy (yes vs. no), was reported at the end of the first trimester. Parental feeding behavior was assessed with three subscales of the CFQ [11], i.e. Monitoring (3 items), Restriction (8 items), and Pressure to Eat (4 items). Examples of items are “How much do you keep track of the high fat foods your child eats?” (Monitoring), and “I intentionally keep some foods out of my child’s reach” (Restriction). The CFQ items are scored on a 5-point Likert scale from 1 ‘never’ to 5 ‘always’. Continuous scale scores were expressed as standard deviation scores to facilitate interpretation. Earlier research provided support for the validity of the CFQ [11,29,30]. Reliability of the CFQ-scales in our sample was moderate (α = .66, Pressure to Eat, *N* = 4743, 4 items) to high (α = .92, Monitoring, *N* = 4766, 3 items).

#### ***Food intake***

When the child was 14 months old, parents completed a food frequency questionnaire (FFQ) to assess children’s food intake. The questionnaire was based on a previously developed and validated FFQ [31], which was adapted on the basis of foods frequently consumed among young children and then validated against 24 h-recalls in a representative sample of children aged 14 months, as described previously [32]. For this validation intracorrelation coefficients were calculated for macronutrients: 0.4 for total energy, 0.7 for total protein, 0.4 for total fat, 0.4 for carbohydrates, and 0.7 for dietary fiber. Parents were asked to indicate how often their child consumed 211 different food items over the past 4 weeks. Following the approach of Kiefte-de Jong and colleagues [33], the food items were classified into 21 food groups (Additional file 3: Table S3). Previous studies indicated that fussy/picky eaters – depending on the definition – eat less fruit, vegetables, grains, meat, and fish than non-fussy/picky eaters [6,12,34,35] as well as less mixed dishes such as many pasta dishes [3]. Some studies also suggest differences in the intake of sweet and fat foods [3,8,12], although the direction of these differences is not clear. In the current study, we included the following food groups: refined grain products (e.g. white bread), wholegrain products (e.g. muesli), dairy products (e.g. yoghurt), formula feeding, staple food (pasta, rice, and potatoes), vegetables (excluding legumes), fruit, fish/seafood (including both fish and shellfish), meat, confectionary (e.g. chocolate), savory snacks (e.g. potato-chips/crisps), and composite dishes. Because we were interested in the relative rather than total amount that was consumed of each foodgroup, the FFQ scores (grams per day) were transformed into z-scores based on the current study population.

### Statistical analyses

To identify eating behavior profiles, we conducted a latent profile analyses (LPA) in Mplus [36] using continuous, z-standardized scores on the CEBQ subscales. Based on our findings from the correlational structure analyses as described above, we decided to exclude the two emotional eating scales and desire to drink from the latent profile analysis. This decision was supported by conceptual considerations: emotional under- and overeating might indicate more of an emotional eating component rather than general food approach or avoidance [37]. Desire to drink was excluded also because it indicates drinking rather than eating behavior, which may conceptually be different. Thus, five scales (FR, EF, SR, SE and FF) remained that were used in the LPA.

Similar to a traditional cluster analysis and latent class analysis, LPA identifies clusters of observations with similar values on a cluster variable using a model-based approach with continuous variables [36]. LPA is a so-called “person centered” approach, which means that observations are clustered on subject basis, unlike factor analysis, in which observations are clustered on item basis. LPA has been frequently used, also in the field of eating behavior, e.g. to identify eating disorder phenotypes in a twin cohort study in Australia [38]. We determined the number of latent profiles based on the minimization of Bayesian information criteria (BIC) [39] and Akaike information criteria (AIC) indices and a non-significant Lo-Mendell-Rubin Likelihood Ratio Test (LMR-LRT) [40] to test model fit. BIC and AIC approaching 0 indicate the best model fit. LMR-LRT indicates whether a solution with an k + 1 profiles fits the data better than a solution with k profiles. Once we decided the optimum number of profiles, assignment of the subjects to one of the profiles was based on Bayesian probabilities.

Subsequently, we examined characteristics of the resulting eating behavior profiles, and compared the group of *fussy* eaters with the non-fussy eaters regarding child and family characteristics as well as food intake at 14 months of age using SPSS 20.0 [41]. Group differences in continuous variables (e.g. food intake, maternal feeding behavior) were tested by independent sample t-tests. Group differences in categorical variables (e.g. parity, ethnicity, sex) were tested with Pearson Chi-Square tests. As a sensitivity analysis, we additionally compared the group of *fussy* eaters to a reference group with average scores regarding these characteristics. Finally, we compared all eating behavioral profiles regarding these background characteristics (data presented as supplementary material) using Multivariate Analysis of Variance (MANOVA), with the average scoring group as reference.

## Results

### Sample characteristics

Characteristics of the study sample are described in Table 1. The majority of children included in the study population were of Western origin (76%) and most (77%) attended daycare for at least 8 hours per week when they were three years old. Mothers were relatively highly educated with more than half having completed at least a college education. Likewise, family income was relatively high with 68% of families earning at least 2200€ per month. Mean maternal BMI before pregnancy 23.3 (SD = 3.9).

**Table 1:** **Sample characteristics (*N*** **= 4914)**

**Child characteristics**		***N***		**Missing *N *** **(%)**
Mean (SD) gestational age at birth (weeks)		4896	39.8 (1.8)	18 (0.3)
Mean (SD) birth weight (g)		4609	3441 (568)	305 (6.2)
Mean (SD) BMI around 4 years^1^		3117	15.8 (1.3)	1797 (36.6)
Sex	% girl	2458	50.0	0
Ethnicity	% non-Western	1188	24.4	39 (0.7)
Firstborn	% yes	2789	56.8	0
Only child at age 4 years	% yes	1042	21.6	82 (1.7)
Daycare attendance at 3 years	% at least 8 h/week	3338	77.1	582 (11.8)
**Family characteristics**				
Mean (SD) maternal age at enrolment (years)		4914	31.5 (4.6)	0
Mean (SD) BMI mother before pregnancy		3703	23.3 (3.9)	1211 (24.6)
Mean (SD) BMI partner before pregnancy		3634	25.2 (3.3)	1280 (26.0)
Marital status	% single	370	7.9	237 (4.8)
Household income	% < 2200€/month	1321	32.4	833 (17.0)
Maternal education	% less than college	1979	42.1	210 (4.3)
Smoking during pregnancy	% yes	944	21.4	501 (10.2)

### Non-response analysis

Comparison of children who were included in factor analysis (*N* = 4914) and those who were excluded due to missing data (*N* = 2381) showed several differences: Excluded children were less often firstborn (48.1%) and less often of Western origin (47.1%) than included children (56.8% firstborn, *p* < .001; 75.6% Western origin, *p* < .001). Excluded children were lighter at birth (mean difference = 103 grams, *p* < .001) and were born earlier (mean difference = 0.16 weeks; *t* (7252) = -3.51, *p* < .001) than included children. Compared to parents of included children, parents of excluded children were more often single (22.4% vs. 7.9%, *p* < .001), lower educated (72.6% vs. 42.1%, *p* < .001) and more often had a low family income (63.1% vs. 32.4%, *p* < .001). Also, mothers of excluded children were younger than mothers of included children (mean difference = 2.9 years, *p* < .001). No differences were found in the sex distribution of included and excluded children.

### Latent profile analysis

Latent profile analysis carried out in Mplus with the 5 remaining CEBQ-subscales (FR, EF, SR, FF, SE) indicated 6 distinct eating behavioral profiles. Although model fit criteria (AIC, BIC) kept decreasing beyond 6 profiles, the Lo-Mendell-Rubin Likelihood Ratio Test was no longer significant in the 7 profile solution (LMR-LRT = 224, *p* = .08), which indicates that the 6 profile solution is the optimal model for our data. Participants were assigned to one of the 6 profiles based on the highest probability of profile membership.

**Figure 1:**
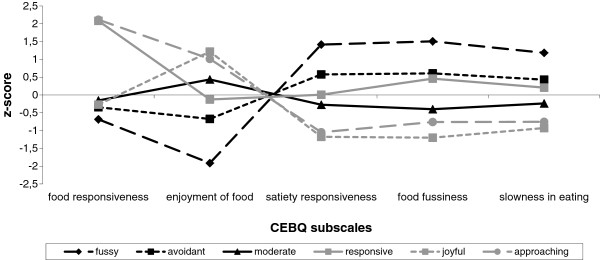
Child eating behavior questionnaire mean subscale scores (z-standardized) in different eating behavior profiles.

Figure 1 shows the pattern of CEBQ scores for each of the six identified eating behavior profiles, among which a distinct “*fussy* eater” profile. The *“fussy* eater*”* profile (5.6% of children) is characterized by a pattern of high scores on food avoidance scales (SR, FF, SE) in combination with low scores on the food approach scales, in particular low enjoyment of food. *Fussy* eaters scored almost 1SD below the mean on FR, and even 2SD lower on EF, and about 1.5 SD higher than the mean on the food avoidance scales (SR, FF, and SE).

The remaining five profiles were: 1) a “*moderate* eater” profile (44.6% of children), characterized by scores around the mean on all included subscales, 2) an *“avoidant* eater*”* profile (33.2%), which is a milder version of the *fussy* profile, with lower than average scores on food approach and higher than average scores on the food avoidance scales, 3) a *“responsive* eater*”* profile (3.9%) with a pattern of very high scores on food responsiveness in combination with average scores on all other scales, 4) a *“joyful* eater*”* profile (5.6%) characterized by low scores on food avoidance and high enjoyment of food, but average food responsiveness, and 5) an *“approaching* eater*”* profile (7.1%) with the opposite pattern of “*fussy* eaters”, i.e. high scores on the food approach scales in combination with low scores on food avoidance scales. Noticeably, the different eating behavior profiles appeared to be mainly discriminated by different patterns of scores on the food approach scales (e.g. high FR/average EF in *responsive* eaters, average FR/high EF in *joyful* eaters, low FR/ low EF in *fussy* eaters) whereas scores on the three food avoidance scales differed not in pattern but in level (all average in *responsive* eaters, all low in *joyful* eaters, all high in *fussy* eaters). Mean scores on the CEBQ subscales per group are given in the supplementary tables (Additional file 4: Table S4).

### Characteristics of *fussy* eaters

We focus on the description of the *fussy* eating behavior profile (*N* = 277) in comparison with non-fussy eaters (*N* = 4638). Characteristics of all six eating behavior profiles we identified can be found in the supplementary tables (Additional file 4: Table S4, Additional file 5: Table S5, Additional file 6: Table S6).

#### ***Child and family characteristics***

*Fussy* eaters differed from non-fussy eaters in several characteristics (see Table 2). *Fussy* eaters were less often girls than non-fussy eaters (43% vs. 50%, *p* < .05) and more often of non-Western origin (32% vs. 24%, *p* < .05). Weight at birth was lower in *fussy* eaters than in non-fussy eaters (mean difference = 72 g, *p* < .01) and at 4 years of age *fussy* eaters still had a lower mean BMI (mean difference = 0.39, *p* < .01) and were more often underweight (19% vs. 12%, *p* < .05).

**Table 2:** Child and family characteristics of fussy and non-fussy eaters

				**Fussy eaters**	**Non-fussy eaters**			
**Child characteristics**		***N *** **for analysis**	***N *** **(%) fussy eaters**	***Mean (SEM)***	***Mean (SEM)***	***t***	***df***	***p***
Gestational age at birth (weeks)		4896	275 (5.6)	39.92 (0.11)	39.84 (0.03)	-0.75	4894	.45
Birth weight (g)		4909	276 (5.6)	3374 (34.2)	3446 (8.3)	2.03	4907	.04
BMI child around 4 y		3117	181 (5.8)	15.45 (0.09)	15.84 (0.03)	3.82	3115	<.01
BMI (SDS) child around 4 y^1^		3117	181 (5.8)	-0.18 (0.07)	0.10 (0.02)	3.90	3115	<.01
								
				**%**	**%**	***Χ***^***2***^	***df***	***p***
Sex	Girl	4914	277 (5.6)	43.3	50.4	5.27	1	.02
Birth weight	Low (<= 2500 g)	4909	276 (5.6)	5.1	4.9	0.01	1	.91
Child ethnicity	Non-Western	4875	270 (5.5)	31.5	24.0	7.85	1	.01
Firstborn	Yes	4914	277 (5.6)	53.8	56.9	1.04	1	.31
Only child at 4 years	Yes	4832	271 (5.6)	23.2	21.5	0.48	1	.49
Daycare attendance at 3 years	No	4332	241 (5.6)	7.1	6.0	2.34	2	.31
	Yes, < 8 h/week			19.9	16.7			
	Yes, ≥ 8 h/week			73.0	77.3			
Weight status 4 years^2^	Underweight	3117	181 (5.8)	19.3	12.3	8.02	2	.02
	Normal weight			73.5	78.6			
	Overweight/obese			7.2	9.1			
								
**Family characteristics**				***Mean (SEM)***	***Mean (SEM)***	***t***	***df***	***p***
Age mother at intake (years)		4914	277 (5.6)	30.82 (.28)	31.55 (.07)	2.55	4912	.01
Maternal feeding behavior	Monitoring, z-score	4862	274 (5.6)	-.24 (.06)	.02 (.02)	3.51	295.81	<.01
	Restricting, z-score	4862	274 (5.6)	-.008 (.06)	-.002 (.02)	0.19	302.04	.85
	Pressuring, z-score	4862	274 (5.6)	.77 (.06)	-.04 (.01)	-18.00	340.17	<.01
BMI mother before pregnancy		3703	207 (5.6)	23.11 (3.5)	23.31 (3.9)	0.70	3701	.49
BMI partner at intake		3634	186 (5.1)	25.17 (3.4)	25.18 (3.3)	0.03	3632	.97
								
				**%**	**%**	***Χ***^***2***^	***df***	***p***
No. of overweight parents (BMI > 25)	1	4255	235 (5.5)	44.3	42.4	0.85	2	.66
	2			8.1	9.8			
At least 1 underweight parent (BMI <18.5)		4910	277 (5.6)	3.6	3.0	0.38	1	.54
Marital status	Single	4677	255 (5.5)	8.6	7.9	0.19	1	.66
Family income (€/month)	< 2200	4081	224 (5.5)	42.0	31.8	9.97	1	<.01
Educational level mother	Less than college	4704	253 (5.4)	54.5	41.4	17.07	1	<.01
Smoking during pregnancy	Yes	4413	242 (5.5)	24.4	21.2	1.36	1	.24

*Note*: results from independent sample t-tests (continuous variables) and chi-square tests (categorical variables). *SEM* = standard error of mean. *N* = number of observations, df = degrees of freedom, *t* = t-value, Χ^2^ = Chi-square-value, *p* = probability for two-sided tests. SDS BMI is standardized to the Dutch population, z-scores are standardized to the population for analysis. ^1^[25,26]^2^[27,28].

Families of *fussy* eaters more often had a lower socio-economic status than families of non-fussy eaters, i.e. mothers had a lower education and family income was lower. Also, mothers of *fussy* eaters were somewhat younger than mothers of non-fussy eaters (mean difference = 0.7 years, *p* < .01). Finally, we found differences in feeding behavior between mothers of *fussy* and non-fussy eaters. Mothers of *fussy* eaters used less monitoring of their children’s eating behavior (mean difference = 0.26 SD, *p* < .01) but more pressure to eat than mothers of non-fussy eaters (mean difference = 0.81 SD, *p* < .01).

#### ***Food intake at age 14 months***

As shown in Table 3, differences in food intake at 14 months of age were found between *fussy* and non-fussy eaters. Children identified as *fussy* eaters when they were four years old, ate less whole grain products (mean difference = 0.28 SD, *p* < .01), less vegetables (mean difference = 0.20 SD, *p* < .05), less fish/seafood (mean difference = 0.16 SD, *p* < .05), and less meat (mean difference = 0.22 SD, *p* < .05) at 14 months of age than children later identified as non-fussy eaters. By contrast intake of savory snacks (mean difference = 0.10 SD, *p* < .05) and confectionary (mean difference = 0.19 SD, *p* < .05) at 14 months was higher in later *fussy* eaters than in non-fussy eaters. There were no significant differences in the intake of the remaining food groups or in total energy intake.

**Table 3:** Dietary intake, BMI and maternal feeding behavior in fussy and non-fussy eaters

	**Fussy eaters *N *** **= 148**	**Non-fussy eaters *N *** **= 2862**			
**Intake, z-score**	***Mean (SEM)***	***Mean (SEM)***	***t***	***df***	***p***
Refined grains	.05 (.08)	-.50 (.02)	-1.31	3008	.19
Whole grains	-.20 (.08)	.08 (.02)	3.35	3008	.00
Dairy	-.13 (.08)	.01 (.02)	1.68	3008	.09
Formula	.07 (.08)	-.01 (.02)	-0.88	3008	.38
Pasta/rice/potatoes	-.16 (.08)	-.02 (.02)	1.79	3008	.07
Vegetables (excl. legumes)	-.21 (.08)	-.01 (.02)	2.42	3008	.02
Fruits (excl. juices)	.03 (.08)	.04 (.02)	0.14	3008	.89
Fish/seafood (excl. fishfingers)	-.16 (.08)	.00 (.02)	2.40	169.77	.02
Meat (excl. savory snacks)	-.18 (.08)	.04 (.02)	2.55	3008	.01
Savory snacks	.16 (.07)	.06 (.02)	-2.03	153.69	.04
Confectionary	.15 (.08)	-.04 (.02)	-2.30	3008	.02
Ready-to-eat	.22 (.08)	.01 (.02)	-1.93	156.06	.06
Sugar sweetened beverages	-.05 (.08)	-.01 (.02)	0.53	160.14	.59
Total energy intake (kcal)	1300 (32.3)	1316 (7.3)	0.50	2997	.62

*Note*: Results of independent sample t-tests (continuous variables) and chi-square tests (categorical variables). *SEM* = standard error of mean, *N* = number of observations, *df* = degrees of freedom, *t* = t-value, Χ^2^ = Chi-square-value, *p* = probability for two-sided tests. Scores were z-standardized to the population of analysis.

### Sensitivity analysis

Because the non-fussy eater group also contained potentially problematic eaters (e.g. *approaching* eaters) we additionally compared fussy eaters to moderate eaters, i.e. a group of children (44.6%) with average scores on all five CEBQ subscales included in the LPA (see Additional file 4: Table S4). Differences between *fussy* and *moderate* were very similar to the difference between *fussy* and non-fussy eaters as a whole. Compared to *moderate* eaters, *fussy* eaters had a lower BMI, were more often underweight, and had mothers who used less monitoring but more pressure to eat (see Additional file 5: Table S5). Likewise, *fussy* eaters more often came from families with lower SES than *moderate* eaters. Differences in consumption of different food groups were also similar to the differences we observed between *fussy* and non-fussy eaters as a whole (see Additional file 6: Table S6). Additionally, *fussy* eaters consumed less staple food (*p* < .05) and more ready-to-eat meals (*p* < .05) than *moderate* eaters.

## Discussion

Using a latent profile approach to identify eating behavior profiles based on the Child Eating Behavior Questionnaire (CEBQ) [14] in 4 year-olds, we found a distinct *fussy* eating behavior profile characterized by a pattern of low scores on the food approach scales and high scores on the food avoidance scales. The *fussy* eating behavior profile was found in 5.6% of children, similar to Micali and colleagues [17] who report a prevalence of 7.3% in 5–7 year-olds also using a data-driven approach, defining a “picky eating” score by factor analysis. By contrast, studies using a single item approach to assign picky eater status (e.g. “Is your child a picky eater?”) found much higher prevalences, e.g. 21% in a study in 3–5 year-olds [7] and up to 50% of 2 year-olds [3]. Similarly, Dubois and colleagues [10] found that 30% of preschoolers were picky eaters, based on a 3-item assessment.

Besides differences in assessment methods, these differences in prevalence might be partly due to the age of assessment of eating behavior. Previous studies have indicated that the highest incidence of picky eating occurs around the age of 2 years [3]. Most likely, fussy eating behavior at this age is driven by food neophobia, i.e. unwillingness to eat new foods, which is often considered to be a part of fussy/picky eating. For example, the CEBQ includes three (out of six) questions about food neophobia in the FF subscale. Food neophobia prevalence rates are known to peak around the age of 2 years, when children become increasingly mobile, and it is beneficial for them to be suspicious towards new foods, from an evolutionary perspective [6]. For most of the children displaying fussy eating behavior at 2 years of age, this will only be a transient phase in normal development. Although our assessment of eating behavior at age 4 years misses the peak incidence of food fussiness, it is more likely to pick up those children with more persistent eating problems. Beyond the specific differences in eating behavior, *fussy* eaters also differed from non-fussy eaters in several child and family characteristics. For example, indices of low socio-economic status, i.e. below modal household income and lower maternal educational level, were more common the group of *fussy* eaters than in non-fussy eaters. There were more boys in the *fussy* eater group (56%) than in the non-fussy eater group (50%).

The validity of the *fussy* eater profile is supported by differences in the intake of certain food groups when children were 14 months of age. *Fussy* eaters consumed less foods that are generally not very popular with children such as vegetables, wholegrain products, fish and meat, which has also been reported by previous studies [6,12,34,35]. By contrast, the intake of food groups that are generally liked by children, including refined grain products such as soft buns and cornflakes, dairy products such as yoghurt, and fruits was similar in *fussy* and in non-fussy eaters. Interestingly, *fussy* eaters consumed more confectionary such as cookies and also more savory snacks such as potato-chips and fast food than non-fussy eaters. Similar findings were reported by previous studies that assessed food intake and food fussiness at the same time point [8]. Possibly mothers of *fussy* eaters are more permissive in letting their children eat palatable but unhealthy foods to compensate for the lower intake of other foods. This may account for the finding that *fussy* eaters did not have a lower total energy intake than non-fussy eaters at 14 months of age. Nevertheless, when they reached the age of four years, *fussy* eaters had a lower BMI and were more often underweight than non-fussy eaters, which has also been shown in previous studies [10,12,42]. Differences in the intake of certain food groups at 14 months might be explained by early differences in preference indicating that *fussy* eaters were already more picky at the age of 14 months. Alternatively, difference in intake might be due to the lack of access to some food groups such as vegetables and whole grain products, especially in lower SES families, or simply that these foods are not offered to the child by the parents. Together, findings indicate that *fussy* eaters have a (history of) more unhealthy diet and body weight than non-fussy eaters, although we could not test whether the difference we found when children were 14 months old actually persisted because food intake was not assessed again till the age four years.

We also found differences in maternal feeding behavior between *fussy* and non-fussy eaters. Mothers of *fussy* eaters used less monitoring of their child’s eating behavior, and applied more pressure to eat, which also suggests that these children are not eating well by themselves. As also pointed out by Jansen and colleagues [1] parental pressure may be a reaction to children’s difficult eating behavior, but may at the same time also have counterproductive effects on child eating behavior such as lowering the child’s enjoyment of food. The associations between maternal feeding behavior and child eating behavior therefore probably represent bi-directional effects on behavioral patterns that have developed in the course of early childhood [43]. Differences between *fussy* and non-fussy children in BMI cannot be explained solely by extreme opposite scores of the potentially overeating children at the other end of the continuum, because these differences were also apparent when we compared the *fussy* eaters to the *moderate* eaters only.

Our study confirmed the good psychometric properties of the CEBQ (14) and replicated the eight factors of the original questionnaire. These factors accounted for about 68% of the total variance, which is very similar to previous findings [14,21]. The eight subscales showed good internal consistency. The correlational structure was generally as expected, with food approach subscales correlating positively with each other, and food avoidance subscales correlating positively with each other, but negatively with the food approach scales. One exception was the positive correlation between emotional undereating and emotional overeating that has been previously reported within the Generation R Study [1]. Other studies also reported inconsistent findings concerning these two scales. For example, in a study by Micali and colleagues [17], eating more or less in response to emotional distress did not load on any of the five factors they identified in a factor analysis concerning child eating style. A possible explanation is that these two scales describe an emotional eating dimension, which is not part of the food approach - food avoidance continuum. Further research is needed to confirm this hypothesis.

Some limitations of this study should be mentioned. Information about child eating behavior was only available for 67% of the participants who gave consent for the preschool phase of Generation R. As expected and typical in population-based studies, non-responders were potentially more problematic families with lower SES, younger mothers, and more single mothers, which may reduce generalizability of our findings. Most importantly, the eating behavior profiles we identified might be typical for our low risk sample. Future studies should examine, if similar patterns can be identified in different groups.

It is well known that FFQs are not reliable in assessing the exact amount of dietary intake and total energy intake in particular [32], which usually leads to an underestimation of the true association with diet. However, FFQs have been proven suited to assess the relative intake, and conclusions may be drawn concerning higher or lower intake of a certain foodgroup, for example by using standardized scores. For the current study, the exact intake of each food or foodgroup was not relevant, as we aimed to describe differences in preferences between the different eating behavior groups which is based on relative differences. However, results should be interpreted with caution, because the FFQ used in this study was only validated for the Dutch population and not for the ethnic minorities included in this cohort [33]. Also, dietary data was only available for 60% of the participants included in the LPA. Additional analyses showed that again data was more often missing for potentially problematic families with lower income, lower educational level, and more often a non-Western origin. This selection-bias indicates caution in generalizing the differences in dietary intake between fussy and non-fussy eaters to other populations.

In summary, in a large population-based study we identified a fussy eating behavioral profile in 5.6% of 4-year olds, characterized by high food fussiness, high satiety responsiveness and slowness in eating in combination with low enjoyment of food and low responsiveness to food. This *fussy* eater profile provides a more detailed eating behavior profile than single items and at the same time distinguishes different potentially problematic eating behavioral groups. Although the single item approach to ask mothers if they consider their child a picky eater has been shown to predict observed eating behavior to some extent [7] factor analytic approaches indicate that fussy/picky eating is a rather complex combination of different behaviors [17]. Multi-item scales assessing fussy/picky eating behavior provide more detailed information about the specific behaviors that are measured, but the lack of a validated cut-off to classify fussy/picky and non-fussy/picky children limits interpretability of results and clinical implications. This profile approach may be used in future studies to better understand child eating and feeding problems and how they develop and predict later eating behavior. Also, it may eventually lead to a better tool in the diagnosis of eating problems, as fussy/picky eating might be reflected not only by high food fussiness, but rather a combination of worrisome eating behaviors, such as low enjoyment of food, slowness in eating and quickly being full. Future studies might also examine how the eating behavior profiles relate to Avoidant/Restrictive Food Intake Disorders, i.e. non-eating disorder eating disturbances characterized by food avoidance and restriction of the amount or range of food intake, which are proposed to be included in the DSM-5 [43]. Finally, a more detailed profile of problematic eating behaviors also provides a better base for the development of prevention and intervention programs for children with feeding and eating problems that can target more specific eating behaviors which may decrease the risk of nutrient deficiencies.

## Conclusions

The identification of the fussy eating behavior profile described in this study is an important step toward an operative diagnosis of fussy/picky eating with implications for future research and the development of diagnostic tools and interventions.

## Competing interests

A. Tharner, J. C. Kiefte-de Jong, and O.H. Franco work in ErasmusAGE, a center for aging research across the life course funded by Nestlé Nutrition (Nestec Ltd.), Metagenics Inc. and AXA. The authors had final responsibility for design and conduct of the study, collection, management, analysis, and interpretation of the data, and preparation, review or approval of the manuscript. No other authors declare a conflict of interest.

## Authors’ contributions

AT: conception of specific research question, analyzed data or performed statistical analysis, wrote paper, had primary responsibility for final content. PWJ: wrote paper, provided advice concerning children’s eating behavior. JCK: wrote paper, provided advice concerning children’s nutritional intake. HAM: designed research (development of overall research plan child feeding), provided advice concerning children’s nutritional intake. JvdE: analyzed data or performed statistical analysis. VWVJ: designed research (development of overall research plan Generation R, study oversight). AH: designed research (development of overall research plan GenerationR). HT: designed research (development of overall research plan child behavior, specific project conception), provided advice concerning child behaviour and statistical analyses. OHF: designed research (specific project conception, project oversight). All authors read and approved the final manuscript.

## Supplementary Material

Additional file 1: Table S1 Exploratory factor analyses of the Child Eating Behavior Questionnaire. Supplementary table showing results of exploratory factor analysis of the Child Eating Behavior Questionnaire, including factor loadings per item and explained variance per factor. Click here for file

Additional file 2: Table S2 Correlations of Child Eating Behavior Questionnaire subscales. Supplementary table showing correlations between the subscales of the Child Eating Behavior Questionnaire. Click here for file

Additional file 3: Table S3 Definition of food groups. Supplementary table showing the separate food items included in the food groups presented in the manuscript. Click here for file

Additional file 4: Table S4 Mean Child Eating Behavior Questionnaire subscale scores of the six eating behavior profiles. Supplementary table showing the mean CEBQ subscale scores in the six eating behavior profiles found with Latent Profile Analysis. Click here for file

Additional file 5: Table S5 Characteristics of children and their families per eating behavior profiles. Supplementary table showing mean scores (SEM) and% of child and family characteristics in the six identified eating behavior profiles in addition to the table presenting characteristics of fussy vs. non-fussy eaters included in the manuscript. Click here for file

Additional file 6: Table S6 Intake of several food groups, BMI and maternal feeding behavior per eating-behavioral style. Supplementary table showing mean (SEM) intake of food groups in the six identified eating behavior profiles in addition to the table presenting intake of fussy vs. non-fussy eaters included in the manuscript. Click here for file
