# Supplementary material for: Toward an operative diagnosis of fussy/picky eating: a latent profile approach in a population-based cohort
Source: Int J Behav Nutr Phys Act. 2014 Feb 10;11:14. doi: 10.1186/1479-5868-11-14 (PMC3922255; doi:10.1186/1479-5868-11-14)
Supplement: Additional file 1: Table S1 — Exploratory factor analyses of the Child Eating Behavior Questionnaire. Supplementary table showing results of exploratory factor analysis of the Child Eating Behavior Questionnaire, including factor loadings per item and explained variance per factor. [file 1479-5868-11-14-S1.doc]

**Additional file 1**

Table S1 - Exploratory factor analyses of the Child Eating Behavior Questionnaire items.

| Factors and items | | | Loading (rotated) | Original scale |  | Factors and items | | Loading (rotated) | Original scale |
| --- | --- | --- | --- | --- | --- | --- | --- | --- | --- |
| **Factor 1 (23% of variance explained)** | | |  |  |  | **Factor 5 (6% of variance explained)** | |  |  |
| 1 | Eats less when upset | | .84 | EUE |  | 23 | Enjoys new food (r) | .72 | FF |
| 2 | Eats less when angry | | .89 | EUE |  | 24 | Enjoys variety of food (r) | .56 | FF |
| 3 | Eats less when tired | | .56 | EUE |  | 25 | Is interested in new food (r) | .74 | FF |
| 4 | Eats more when happy | | .43 | EUE |  | 26 | Refuses to eat new food at first | .81 | FF |
|  |  | |  |  |  | 27 | Decides not to like food without tasting it | .73 | FF |
| **Factor 2 (13% of variance explained)** | | |  |  |  |  |  |  |  |
| 5 | Eats more when anxious | | .88 | EOE |  | **Factor 6 (5% of variance explained)** | |  |  |
| 6 | Eats more when annoyed | | .86 | EOE |  | *28* | *Difficult to please with meals* | *-.37* | *FF* |
| 7 | Eats more when worried | | .93 | EOE |  | 29 | Enjoys eating | .82 | EF |
| 8 | Eats more when nothing to do | | .46 | EOE |  | 30 | Loves food | .90 | EF |
|  |  | |  |  |  | 31 | Interested in food | .75 | EF |
| **Factor 3 (8% of variance explained)** | | |  |  |  | 32 | Looks forward to mealtime | .65 | EF |
| 9 | Always asks for food | | .48 | FR |  |  |  |  |  |
| 10 | Would always have food in mouth | | .82 | FR |  | **Factor 7 (4% of variance explained)** | |  |  |
| 11 | Would eat most of the time | | .84 | FR |  | 19 | Eats slowly | .80 | SE |
| 12 | Would eat too much | | .86 | FR |  | 20 | Takes more than 30 minutes to finish a meal | .85 | SE |
| 13 | Has always room for favorite meal | | .59 | FR |  | 21 | Finishes meal quickly (r) | .39 | SE |
| *14* | *Has a big appetite (r)* | | *-.39* | *SR* |  | 22 | Eats more and more slowly in the course of a meal | .34 | SE |
|  |  | |  |  |  |  |  |  |  |
| **Factor 4 (6% of variance explained)** | | |  |  |  | **Factor 8 (3% of variance explained)** | |  |  |
| 15 | | Leaves food on the plate | .57 | SR |  | 33 | Would always love drink | .88 | DD |
| 16 | | Is full easily | .73 | SR |  | 34 | Would drink continuously | .89 | DD |
| 17 | | Is full before meal is finished | .88 | SR |  | 35 | Always asks for a drink | .77 | DD |
| 18 | | Cannot eat meal after snack | .38 | SR |  |  |  |  |  |

*Note*: Rotation Method Geomin. CEBQ items were rated on a 5-point Likert scale, analyzed continuously. Factors selected based on Eigenvalue >1 and screeplot. EOE=Emotional Overeating; FR=Food responsiveness; EF=Enjoyment of Food; DD=Desire to Drink; EUE=Emotional Undereating; SR=Satiety Responsiveness; FF=Food Fussiness; SE=Slowness in Eating. “(r)” means this item was reverse coded.
