# Supplementary material for: Toward an operative diagnosis of fussy/picky eating: a latent profile approach in a population-based cohort
Source: Int J Behav Nutr Phys Act. 2014 Feb 10;11:14. doi: 10.1186/1479-5868-11-14 (PMC3922255; doi:10.1186/1479-5868-11-14)
Supplement: Additional file 2: Table S2 — Correlations of Child Eating Behavior Questionnaire subscales. Supplementary table showing correlations between the subscales of the Child Eating Behavior Questionnaire. [file 1479-5868-11-14-S2.doc]

**Additional file 2**

Table S2 - Correlations of Child Eating Behavior Questionnaire subscales

|  | EOE | FR | EF | DD | EUE | SR | FF | SE |
| --- | --- | --- | --- | --- | --- | --- | --- | --- |
| EOE | 1 |  |  |  |  |  |  |  |
| FR | .21 | 1 |  |  |  |  |  |  |
| EF | -.05 | .27 | 1 |  |  |  |  |  |
| DD | .18 | .22 | -.01 | 1 |  |  |  |  |
| EUE | .23 | .03 | -.15 | .05 | 1 |  |  |  |
| SR | .09 | -.14 | -.44 | .09 | .40 | 1 |  |  |
| FF | .00 | -.06 | -.47 | .03 | .23 | .33 | 1 |  |
| SE | .04 | -.13 | -.37 | .09 | .28 | .52 | .20 | 1 |

*Note:* CEBQ subscale scores, max. 2 missings allowed, corrected for number of items endorsed. EOE=Emotional Overeating; FR=Food responsiveness; EF=Enjoyment of Food; DD=Desire to Drink; EUE=Emotional Undereating; SR=Satiety Responsiveness; FF=Food Fussiness; SE=Slowness in Eating. *N* ranges from 4635 for SR and EO and N = 4789 for FF and FR.
