# Supplementary material for: Toward an operative diagnosis of fussy/picky eating: a latent profile approach in a population-based cohort
Source: Int J Behav Nutr Phys Act. 2014 Feb 10;11:14. doi: 10.1186/1479-5868-11-14 (PMC3922255; doi:10.1186/1479-5868-11-14)
Supplement: Additional file 3: Table S3 — Definition of food groups. Supplementary table showing the separate food items included in the food groups presented in the manuscript. [file 1479-5868-11-14-S3.doc]

**Additional file 3**

Table S3 - Definition of food groups.

| **Food group** | **Included food item** |
| --- | --- |
| Refined grain products | Waffles, rusk, crackers, currant bread, currant buns, white buns croissant, white bread or baguette, cornflakes, low fiber breakfast cereal |
| Whole grain products | Whole-bran bread or baguette, whole-bran buns, oatmeal, muesli, multigrain breakfast cereal |
| Dairy | All cheeses, milk (except soy milk), yoghurt, French cheese, custard (excluding chocolate milk or sweetened yoghurt drinks) |
| Formula feeding | All formula feeding |
| Pasta/rice/potatoes | Pasta, rice, and potatoes (boiled, baked or mashed) |
| Vegetables | Vegetables (raw, boiled, or baked) |
| Fruit | Fruit and fruit compote (excluding juice) |
| Fish/seafood | Fish and seafood (excluding fishfingers, which are included in the “savory snacks” category) |
| Meat | All processed and non-processed meat (except meat-containing snacks such as chicken-nuggets which are included in the “savory snacks” category) |
| Confectionary | Dutch spiced honey cake. sweetened or chocolate containing desserts, chocolate containing sandwich spread, ice cream, cakes, cookies, biscuits, chocolate, pastries, pancakes, candy |
| Savory snacks | Chips, toast with cheese or pâté, sausage rolls, spring rolls, meat rolls, meat croquettes, sate, salted peanuts and nuts, hamburgers, chicken nuggets, fried chips and fried potatoes (i.e. French fries) |
| Composite dishes | Ready to eat infant meals, and ready to eat cooled or frozen meals |

*Note*: adapted from Kiefte-de Jong et al. (2012).
