# Supplementary material for: Toward an operative diagnosis of fussy/picky eating: a latent profile approach in a population-based cohort
Source: Int J Behav Nutr Phys Act. 2014 Feb 10;11:14. doi: 10.1186/1479-5868-11-14 (PMC3922255; doi:10.1186/1479-5868-11-14)
Supplement: Additional file 4: Table S4 — Mean Child Eating Behavior Questionnaire subscale scores of the six eating behavior profiles. Supplementary table showing the mean CEBQ subscale scores in the six eating behavior profiles found with Latent Profile Analysis. [file 1479-5868-11-14-S4.doc]

**Additional file 4**

Table S4 - Mean Child Eating Behavior Questionnaire subscale scores of the six eating behavior profiles.

|  | Moderate eaters  (reference) | Fussy  eaters | Avoidant eaters | Joyful  eaters | Responsive  eaters | Approaching  eaters |  |  |  |
| --- | --- | --- | --- | --- | --- | --- | --- | --- | --- |
| Child eating behavior questionnaire subscales, z-score | *Mean(SEM)* | *Mean(SEM)* | *Mean(SEM)* | *Mean(SEM)* | *Mean(SEM)* | *Mean(SEM)* | *F* | *p* | *Partial Eta^2^* |
| Food responsiveness | -0.15 (.01) | -0.71 (.04)** | -0.35 (.02)** | -0.34 (.04)** | 2.20 (.05)** | 2.18 (.03)** | 1491.13 | .00 | .61 |
| Enjoyment of food | 0.47 (.01) | -2.04 (.03)** | -0.73 (.01)** | 1.44 (.03)** | -0.20 (.04)** | 1.04 (.03)** | 2241.91 | .00 | .70 |
| Satiety responsiveness | -0.30 (.02) | 1.48 (.04)** | 0.60 (.02)** | -1.29 (.05)** | -0.01 (.05)** | -1.06 (.04)** | 808.60 | .00 | .46 |
| Food fussiness | -0.42 (.02) | 1.60 (.04)** | 0.65 (.02)** | -1.40 (.04)** | 0.50 (.05)** | -0.80 (.04)** | 1076.96 | .00 | .53 |
| Slowness in eating | -0.26 (.02) | 1.25 (.05)** | 0.46 (.02)** | -1.05 (.05)** | 0.21 (.06)** | -0.74 (.05)** | 391.36 | .00 | .29 |

*Note:* Group differences in mean scores were tested by ANOVA. **p* < .05, ***p* < .01 in pairwise comparisons with the reference group (“*moderate* eaters”). Means are estimated marginal means. *SEM* = standard error of mean. *F* = variance ratio. *P* = probability for two-sided tests.
