# Supplementary material for: Toward an operative diagnosis of fussy/picky eating: a latent profile approach in a population-based cohort
Source: Int J Behav Nutr Phys Act. 2014 Feb 10;11:14. doi: 10.1186/1479-5868-11-14 (PMC3922255; doi:10.1186/1479-5868-11-14)
Supplement: Additional file 5: Table S5 — Characteristics of children and their families per eating behavior profiles. Supplementary table showing mean scores (SEM) and% of child and family characteristics in the six identified eating behavior profiles in addition to the table presenting characteristics of fussy vs. non-fussy eaters included in the manuscript. [file 1479-5868-11-14-S5.doc]

Table S5 - Characteristics of children and their families per eating behavior profiles.

|  | | | | | Moderate eaters (ref) | Fussy  eaters | Avoidant eaters | Joyful  eaters | Responsive  eaters | Approaching  eaters |  |  |
| --- | --- | --- | --- | --- | --- | --- | --- | --- | --- | --- | --- | --- |
| **Child characteristics** | | | | | *Mean (SEM)* | *Mean (SEM)* | *Mean (SEM)* | *Mean (SEM)* | *Mean (SEM)* | *Mean (SEM)* | *F (df)* | *p* |
| Gestational age at birth (weeks) | | | | | 39.85 (.04) | 39.92 (.11) | 39.88 (.05) | 39.62 (.11) | 39.76 (.13) | 39.75 (.10) | 1.43 (5, 4890) | .21 |
| Birth weight (g) | | | | | 3470 (12.1) | 3374 (34.2)** | 3398 (14.1)** | 3441 (34.2) | 3496 (40.9) | 3489 (30.5) | 4.64 (5, 4903) | .00 |
| BMI around 4y | | | | | 15.8 (.04) | 15.5 (.10)** | 15.6 (.04)** | 16.1 (.10)* | 16.5 (.12)** | 16.9 (.09)** | 45.12 (5, 3111) | .00 |
| SDS BMI around 4 y | | | | | 0.11 (.03) | -0.18 (.07)** | -0.10 (.03)** | 0.25 (.07) | 0.50 (.09)** | 0.77 (.06)** | 39.95 (5, 3111) | .00 |
|  | | | | | % | % | % | % | % | % | *Χ^2^(df)* | *p* |
| Sex | | girl | | | 50.5 | 43.3 | 50.3 | 46.4 | 53.1 | 52.2 | 8.07 (5) | .15 |
| Birth weight | | low (<= 2500g) | | | 5.1 | 5.1 | 4.6 | 5.1 | 4.7 | 5.5 | 0.75 (5) | .98 |
| Child ethnicity | | non-Western | | | 21.2 | 31.5** | 25.1** | 27.4* | 33.2** | 28.4** | 31.90 (5) | .00 |
| Firstborn | | yes | | | 56.3 | 53.8 | 58.7 | 53.3 | 52.1 | 58.0 | 7.01 (5) | .22 |
| Only child at 4 years | | yes | | | 19.7 | 23.2 | 22.5* | 26.8** | 17.4 | 25.5* | 15.4 (5) | .01 |
| Daycare attendance at 3 years | | | no | | 5.3 | 7.1 | 5.9 | 10.3 | 6.0 | 7.5 | 17.31 (10) | .07 |
|  | yes, < 8h/week | | | | 16.3 | 19.9 | 16.2 | 19.8 | 18.7 | 17.7 |  |  |
| Weight status 4y | yes, ≥ 8h/week | | | | 78.3 | 73.0 | 77.9 | 69.8 | 75.3 | 74.8 |  |  |
|  | underweight | | | | 10.9 | 19.3* | 16.1 | 10.9 | 7.7 | 5.3 | 166.98 (10) | .00 |
|  | normal weight | | | | 81.4 | 73.5 | 78.5 | 77.0 | 73.5 | 65.2 |  |  |
|  | overweight/obese | | | | 7.8 | 7.2 | 5.4 | 12.1 | 18.8 | 29.5 |  |  |
| **Family characteristics** | | | | | *Mean (SEM)* | *Mean (SEM)* | *Mean (SEM)* | *Mean (SEM)* | *Mean (SEM)* | *Mean (SEM)* | *F(df)* | *p* |
| Age mother at intake (years) | | | | | 31.8 (.10) | 30.8 (.28)** | 31.6 (.14) | 31.1 (.28)* | 30.3 (.33)** | 31.1 (.25)* | 6.45 (5, 4908) | .00 |
| BMI mother at intake | | | | | 24.3 (.09) | 24.4 (.26) | 24.3 (.11) | 24.5 (.26) | 24.9 (.32) | 24.6 (.23) | 0.99 (5, 4368) | .42 |
| BMI partner at intake | | | | | 25.2 (.08) | 25.2 (.24) | 25.1 (.09) | 25.5 (.23) | 25.5 (.29) | 25.3 (.21) | 0.93 (5, 3628) | .46 |
| Maternal feeding behavior | | | | |  |  |  |  |  |  |  |  |
| Monitoring, z-score | | | | | 0.09 (.02) | -0.24 (.06)** | -0.07 (.03**) | 0.17 (.06) | -0.26 (.07)** | 0.04 (.05) | 12.93 (5, 4856) | .00 |
| Restricting, z-score | | | | | -0.08 (.02) | -0.01 (.06) | 0.02 (.02)** | -0.37 (.06)** | 0.51 (.07)** | 0.44 (.05)** | 34.60 (5, 4856) | .00 |
| Pressuring, z-score | | | | | -0.18 (.02) | 0.77 (.06)** | 0.32 (.02)** | -0.48 (.06)** | 0.13 (.07)** | -0.62 (.05)** | 137.78 (5, 4856) | .00 |
|  | | | | | % | % | % | % | % | % | *Χ^2^(df)* | *p* |
| No. of overweight parents (BMI > 25) | | | | 0 | 47.7 | 47.7 | 48.6 | 47.3 | 42.7 | 48.5 | 5.78 (10) | .83 |
|  | | | | 1 | 42.6 | 44.3 | 42.1 | 43.6 | 46.5 | 39.3 |  |  |
|  | | | | 2 | 9.7 | 8.1 | 9.4 | 9.1 | 10.8 | 12.2 |  |  |
| ≥1 parent underweight (BMI <18.5) | | | | | 3.3 | 4.3 | 3.4 | 4.1 | 2.5 | 4.0 | 1.58 (5) | .90 |
| Marital status | | | single | | 6.9 | 8.6 | 7.8 | 10.2 | 8.9 | 11.8** | 12.28 (5) | .03 |
| Family income (€/month) | | | < 2200 | | 29.4 | 42.0** | 32.2 | 37.4* | 37.7* | 38.1** | 25.97 (5) | .00 |
| Educational level mother | | | not high | | 38.3 | 54.5** | 41.4 | 51.7** | 52.0** | 46.4** | 48.43 (5) | .00 |
| Smoking during pregnancy | | | yes | | 19.4 | 24.4 | 22.6* | 23.4 | 15.9 | 27.0** | 16.94 (5) | .00 |

*Note:* Group differences in mean scores were tested by analyses of variance or multivariate analyses of variance (maternal feeding behavior). Group differences in categorical variables were tested by Pearson Chi-Square. **p* < .05, ***p* < .01 in pairwise comparisons with the reference group (“*moderate* eaters”). Means are estimated marginal means. *SEM* = standard error of mean. *F* = variance ratio. *P* = probability for two-sided tests. *N* = number of observations. *N* = 4081 for family income and *N* = 4914 for firstborn and gender. *N* per profile may vary slightly in the different analyses.
