# Supplementary material for: Toward an operative diagnosis of fussy/picky eating: a latent profile approach in a population-based cohort
Source: Int J Behav Nutr Phys Act. 2014 Feb 10;11:14. doi: 10.1186/1479-5868-11-14 (PMC3922255; doi:10.1186/1479-5868-11-14)
Supplement: Additional file 6: Table S6 — Intake of several food groups, BMI and maternal feeding behavior per eating-behavioral style. Supplementary table showing mean (SEM) intake of food groups in the six identified eating behavior profiles in addition to the table presenting intake of fussy vs. non-fussy eaters included in the manuscript. [file 1479-5868-11-14-S6.doc]

**Additional file 6**

Table S6 - Intake of several foodgroups per eating-behavioral style

|  | Moderate eaters  (ref)  *n* = 1390 | Fussy  eaters  *n* = 148 | | Avoidant  eaters  *n* = 970 | | Joyful  eaters  *n* = 167 | | Responsive  eaters  *n* = 116 | | Approaching  eaters  *n* = 219 | |  |  |
| --- | --- | --- | --- | --- | --- | --- | --- | --- | --- | --- | --- | --- | --- |
| Intake, z-score | *Mean (SEM)* | *Mean (SEM)* | | *Mean (SEM)* | | *Mean (SEM)* | | *Mean (SEM)* | | *Mean (SEM)* | | *F* (5, 3004) | *p* |
| Refined grains | -.04 (.03) | .05 (.08) |  | -.10 (.03) |  | .05 (.07) |  | .03 (.09) |  | -.02 (.06) |  | 1.51 | .183 |
| Whole grains | .11 (.03) | -.20 (.08) | ** | .01 (.03) | * | .05 (.08) |  | .13 (.09) |  | .15 (.07) |  | 3.78 | .002 |
| Dairy | .03 (.03) | -.13 (.08) |  | -.02 (.03) |  | -.03 (.08) |  | .13 (.09) |  | .02 (.07) |  | 1.27 | .275 |
| Formula | .03 (.03) | .07 (.08) |  | -.003 (.03) |  | -.15 (.08) |  | -.11 (.09) |  | -.06 (.07) |  | 1.61 | .153 |
| Pasta/rice/potatoes | .02 (.03) | -.16 (.08) | * | -.13 (.03) | ** | .13 (.07) |  | -.03 (.09) |  | .12 (.06) |  | 5.32 | .000 |
| Vegetables (excl. legumes) | .05 (.03) | -.21 (.08) | ** | -.14 (.03) | ** | .10 (.08) |  | -.02 (.09) |  | .11 (.07) |  | 6.80 | .000 |
| Fruits (excl. juices) | .08 (.03) | .03 (.08) |  | -.01 (.03) |  | .02 (.08) |  | .04 (.09) |  | .10 (.07) |  | 1.23 | .294 |
| Fish/seafood (excl. fishfingers) | .04 (.03) | -.16 (.08) | * | -.08 (.03) | ** | .10 (.07) |  | .12 (.09) |  | -.01 (.06) |  | 3.43 | .004 |
| Meat (excl. savory snacks) | .10 (.03) | -.18 (.08) | ** | -.07 (.03) | ** | -.07 (.08)8 | * | .06 (.09) |  | .15 (.07) |  | 5.33 | .000 |
| Savory snacks | -.08 (.02) | .16 (.07) | ** | -.10 (.03) |  | -.02 (.07) |  | .02 (.08) |  | .13 (.06) | ** | 4.43 | .001 |
| Confectionary | -.04 (.03) | .15 (.08) |  | -.04 (.03) |  | -.09 (.07) |  | .09 (.09) |  | -.01 (.07) |  | 1.60 | .157 |
| Ready-to-eat | -.01 (.03) | .22 (.08) |  | .05 (.03) |  | -.06 (.08) |  | -.06 (.09) |  | .05 (.07) |  | 1.98 | .078 |
| Sugar sweetened beverages | .01 (.03) | -.05 (.08) |  | -.08 (.03) | *** | .13 (.08) |  | .21 (.09) | *** | .00 (.07) |  | 2.92 | .012 |
|  |  |  |  |  |  |  |  |  |  |  |  |  |  |
|  | *n* = 1386 | *n* = 146 |  | *n* = 969 |  | *n* = 165 |  | *n* = 115 |  | *n* = 218 |  | *F* (5, 2993) | *p* |
| Total energy intake | .04 (.03) | -.06 (.08) |  | -.13 (.03) | ** | .004 (.07) |  | .08 (.09) |  | .09 (.06) |  | 4.38 | .001 |

*Note*: results of multivariate analyses of variance with eating-behavioral style entered as fixed factor. Means are estimated marginal means. *SEM* = standard error of mean. *F* = variance ratio. *P* = probability for two-sided tests. *N* = number of observations. *N* differed per variable assessed. **p* < .05, ***p* < .01 in pairwise comparisons with the reference group (“*moderate* eaters”).
